# Fabrication of EPYR/GNP/MWCNT carbon-based composite materials for promoted epoxy coating performance

**DOI:** 10.1039/c8ra03109f

**Published:** 2018-06-28

**Authors:** Mahmoud A. Hussein, Bahaa M. Abu-Zied, Abdullah M. Asiri

**Affiliations:** Chemistry Department, Faculty of Science, King Abdulaziz University P. O. Box 80203 Jeddah 21589 Saudi Arabia maabdo@kau.edu.sa mahussein74@yahoo.com; Chemistry Department, Faculty of Science, Assiut University Assiut 71516 Egypt mahmali@aun.edu.eg; Center of Excellence for Advanced Materials Research (CEAMR), King Abdulaziz University P. O. Box 80203 Jeddah 21589 Saudi Arabia

## Abstract

The present study is aimed to fabricate composite materials containing epoxy resin (EPYR) reinforced by mixed carbon-based nano-fillers in the form of graphene nano-platelet (GNP) and multi-walled carbon nanotube (MWCNT) using the dissolution casting technique with the help of ultrasonic assistance. The pure epoxy resin was reinforced by variable loading of mixed GNP/MWCNT *in situ*, and the epoxy resin is denoted as EPYR/GNP/MWCNT_2–30_. The numbers 2–30 corresponded to the final mass ratio of the nano-fillers. The designed products were reinforced by variable percentages of GNP/MWCNTs. XRD, FT-IR, thermal analyses, FE-SEM, TEM and electrical conductivity were utilized as identification techniques to confirm the structures of these composite materials. An excellent evidence for the composite formation was given by XRD diffraction patterns and FT-IR spectroscopy. The introduced amounts of mixed nano-fillers showed significant effects on the thermal, conducting and coating behaviors of pure EPYR. Pure EPYR and EPYR/GNP/MWCNT_20,30_ showed higher thermal stabilities than other materials in the range of 400–410 °C. EPYR/GNP/MWCNT_20_ also showed remarkable increase in the thermal stability compared to other materials. *T*_10_ represents the temperatures at which 10% weight losses are examined. Pure EPYR and its related EPYR/GNP/MWCNT_2–30_ displayed similar thermal stabilities at *T*_10_ temperature (330 ± 4 °C). The morphological features were examined by SEM and TEM; these features showed that the nanocomposite components were extremely compatible. The *in situ* electrical conductivity values showed noticeable enhancement for the formulations of EPYR/GNP/MWCNT_2–10_. Moreover, the coating performance of EPYR was tested by water uptake experiments and electrochemical impedance; both tests proved that the mixed GNP/MWCNT nano-fillers remarkably improved the pure EPYR coating due to the ionic charge transfer resistance and elevated barrier behaviour. The coating resistance variations values (CRv) of EPYR/GNP/MWCNT_10_ were the highest among the measured composition values, closely followed by those of EPYR/GNP/MWCNT_20_ and EPYR/GNP/MWCNT_30_.

## Abstract 2

Composite materials containing epoxy resin and reinforced by mixed carbon-based nano-fillers (GNP/MWCNT) have been fabricated using the dissolution casting technique with the help of ultrasonic assistance for promoted epoxy coating performance.

## Graphical abstract

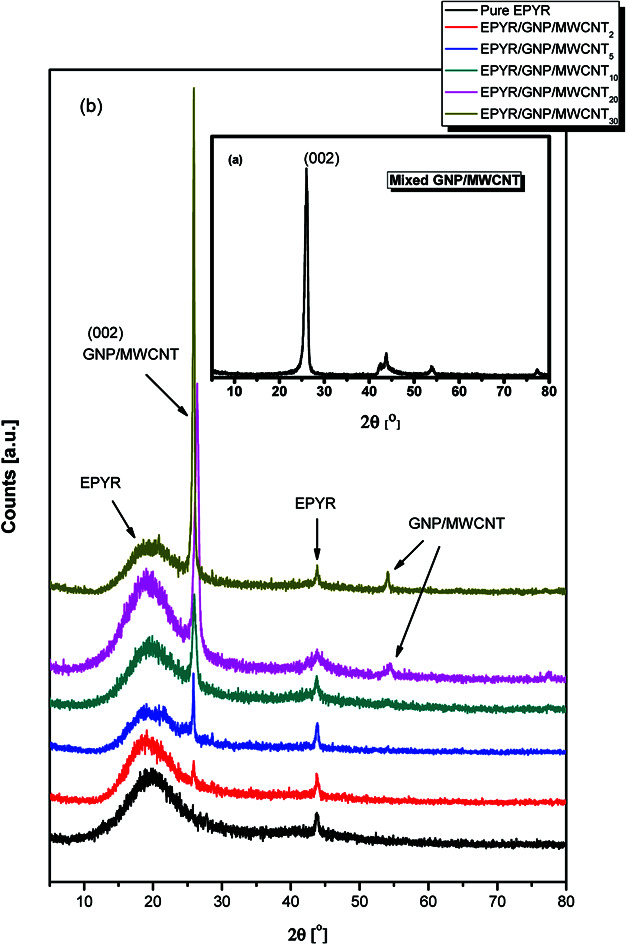

## 1. Introduction

Polymer composite materials are classified as high-performance products that can be produced by a variety of techniques. The broad range of applications of the polymer composite materials and their considerable properties have been given considerable attention in the past few decades. Polymer composite materials have also been frequently distinguished, and they display fundamental properties due to their low cost and many ameliorations in their complete performances;^1–7^ other properties such as better mechanical performance, high operating temperature range, electrostatic discharge, and sufficient chemical resistance should also be studied.^8^ More particularly, the polymer matrix can exhibit significant properties because of nano-filler loading. The main reason for such improvements is the presence of nano-filler reinforcement agents in the form of nano-particles; the insertion of these particles is frequently coveted due to the fact that it can affect the major properties of the used polymer matrix, which include manifestation, processability and weight. Thermal, mechanical, bulkhead, flame properties and many other properties of polymer composite materials have proved the significant effects of such materials.^9–15^ Many nano-fillers have been reported in the literature as reinforcement agents; these include calcium carbonate, alumina, silica, nanoclay, titania, and nanocarbon materials (carbon nanotube and graphene). These reinforcement agents improve almost all the physical and chemical properties, thus preventing nimbleness, shortage of embrittlement and absence of transparency.^16–18^ A considerable effect on the eventual behaviors of composite materials basically arises from excellent cohesion between the polymer matrix and its nano-filler reinforcement agents, due to which the applied fatigue is transferred from the persistent matrix to nano-filler. Till now, many researchers are still designing new carbon nanoparticles and discussing their assistance for enhancing various properties of different polymer matrices in the form of composite new materials. Carbon based materials, for example, carbon fibers (CF), carbon nanotube (CNT) expanded graphite (EG) and functionalized graphene sheets (FGS) are considered as engaging reinforcements that can be easily reinforced in different matrices; they have been widely examined due to their extended as well as excellent electrical, thermal, mechanical, and tribological properties. Besides, they are favorable candidates for the reinforcement of polymer matrices.^19–21^ In the open literature, considerable research papers have previously elucidated the positive impacts of different forms of individual CNT or graphene on pure epoxy resins.^20,22^ The significant effect of mixing CNT and graphene as a mixed reinforcement agent into PANI as a polymer matrix has been recently reported.^21^ In addition, no studies have reported epoxy resin-reinforced mixed CNT and graphene. Therefore, as a continuation of our previous study,^23^ the present manuscript is directed to fabricate a new category of epoxy resin composite materials reinforced by mixed graphene nano-platelet/carbon nanotube. The desired mixed nanofiller GNP/MWCNT was prepared first using an ultrasonicator. EPYR/GNP/MWCNT_2–30_ have been fabricated using variable loadings of previously prepared mixed GNP/MWCNT as mixed reinforcements, whereas epoxy resin is prepared in chloroform, which is an easily evaporated organic solvent. Such fabricated products are estimated and characterized by common techniques, which include X-ray diffraction analysis, Fourier transform infrared spectroscopy (FT-IR), field emission scanning electron microscopy and transmission electron microscopy. Furthermore, the effect of GNP/MWCNT on the enhanced performance of pure EPYR is tested by thermal analyses, electrical conductivity, sorption of water and electrochemical impedance measurements.

## 2. Experimental

### 2.1. Materials and chemicals

Commercially obtained Epikote-1001 x-75% (2642) epoxy together with crayamid – 100% (2580) epoxy hardener was applied as pure epoxy resin; they were also used as obtained without additional purification. Epikote : crayamid (1 : 1) weight by weight was adjusted as exact mixing ratio for pure epoxy processing and fabrication. Spectroscopic grade chloroform was obtained from Sigma-Aldrich, and it was also used without any additional purification. Furthermore, multi-walled carbon nanotubes (CNTs) with an average diameter of 110.0–170.0 nm were purchased from Sigma-Aldrich, and they were used as received without any purification. More particularly, graphene nano-platelet (GNP) was also purchased from Sigma-Aldrich and used as received. Any other chemicals or materials used were of high purity and were also obtained from known sources; they were used as obtained without further purifications, including spectroscopic grade chloroform from Sigma-Aldrich.

### 2.2. Neat epoxy resin film preparation

A thin film of neat epoxy resin was readily fabricated using the casting method and ultrasonic assistance.^23–25^ The following procedures were applied: in 50 mL beaker, a fixed weight of 1 g of Epikote-1001 was dissolved in 25 mL of chloroform. One g of crayamid hardener was also dissolved in 25 mL of chloroform in another beaker. In a closed container, both solutions were directed to an ultrasonicator for 10 minutes before mixing together. Then, the total mixture was permanently exposed to the ultrasonicator for another 10 minutes. This sonicated mixture was poured carefully into a Petri dish and left overnight at room temperature for solvent evaporation. The neat epoxy thin film was collected easily and dried in the oven at 40 °C.

### 2.3. Mixed GNP/MWCNT nano-filler preparation

Mixed nano-filler-based GNP/MWCNT was prepared using the typical procedure as follows:^21^ a mixture of GNP and MWCNTs was first dispersed in distilled water with a mass ratio of 40 to 60% for GNPs to CNTs, respectively. The dispersion was then sonicated for 1 hour using an ultrasonicator. Finally, the mixture was filtered off, dried at 100 °C for 24 hours and kept ready for the next experiments.

### 2.4. EPYR/GNP/MWCNT composite material fabrication

A series of composite materials with a general formula of EPYR/GNP/MWCNT_2–30_ was frugally fabricated based on the above-mentioned neat epoxy film procedures. The expected products were fabricated *in situ*, and the perspicuous epoxy resin was introduced. Such a mixture was considered to have a fixed mixed ratio of variable loading inside the EPYR polymer matrix. We applied 2%, 5%, 10%, 20% and 30% loadings of previously prepared mixed GNP/MWCNT as reinforcement agents in the fabrication process. In an ideal procedure, EPYR/GNP/MWCNT composite materials were fabricated as follows: variable weights of GNP/MWCNT, 1 g of Epikote-1001 and 1 g of crayamid hardener were separately dissolved in 20 mL of chloroform and were exposed to ultrasonicator for 10 minutes. A mixture of these three components was continuously exposed to ultrasonicator for additional 10–15 minutes in a closed container. The sonicated mixture was poured carefully into a Petri dish and left overnight at room temperature for solvent evaporation. A thin film of EPYR/GNP/MWCNT was separated out easily each time and dried in the oven at around 40–50 °C. These procedures were repeated five times by introducing different weights of GNP/MWCNT each time. The designed compositions for EPYR/GNP/MWCNT formulations are illustrated in Table 1.

**Table 1:** Given abbreviations and chemical compositions for pure EPYR and its corresponding EPYR/GNP/MWCNT_2–30_ composite materials

Abbreviation	EPYR (weight, g)	GNP : MWCNT (%)	Mixed GNP/MWCNT loading %, (weight, g)
Pure EPYR	(2 g)	—	—
EPYR/GNP/MWCNT_2_	(1.96)	40 : 60	2%, (0.04)
EPYR/GNP/MWCNT_5_	(1.90)	40 : 60	5%, (0.10)
EPYR/GNP/MWCNT_10_	(1.80)	40 : 60	10%, (0.20)
EPYR/GNP/MWCNT_20_	(1.60)	40 : 60	20%, (0.40)
EPYR/GNP/MWCNT_30_	(1.40)	40 : 60	30%, (0.60)

### 2.5. EPYR/GNP/MWCNT fabricated electrodes

Modified electrodes were fabricated *in situ*, whereas the composite materials were produced according to different loadings of mixed GNP/MWCNT. Working electrodes in the form of EPYR/GNP/MWCNT_2–30_ were based on stainless steel sheets with dimensions of 1 × 1 cm^2^. To obtain good modified electrodes as previously mentioned, EPYR/GNP/MWCNT_2–30_ composite materials were prepared using similar procedures and simultaneously delivered onto the stainless steel sheets in a dropwise manner. These electrodes were cured overnight and then dried for 3–6 h in the oven at temperatures of 40–50 °C. The electrodes were kept dry until their further use in the measurements of coating performance of our composite materials by electrochemical impedance spectroscopy.

### 2.6. Characterization and identification techniques

Powder X-ray diffractograms were determined in the 2*θ* range from 5 to 80° with the aid of Philips diffractometer (type PW 103/00) using the Ni-filtered CuKα radiation. FT-IR spectra were examined by using the ATR smart part technique in the wave number range 4000–400 cm^−1^ using a Thermo-Nicolet-6700 FT-IR spectrophotometer. TGA curves for thermal analysis were recorded with a TA instrument apparatus model TGA-Q500 using a heating rate of 10 °C min^−1^ under nitrogen atmosphere over the temperature range of 20–800 °C. The average masses of the samples were 5–10 mg. The morphological features were characterized by a field emission scanning electron microscope (Jeol-JSM-5400 LV-SEM, Japan). The SEM samples were prepared by evaporating a dilute solution of each nanocomposite on a smooth surface of aluminum foil and subsequently coating it with gold palladium alloy. The microscope was operated at an accelerating voltage of 15 kV and 4 mm work distance carbon film. Transmission electron microscopy (TEM) was conducted using JEOL JEM-1230 operating at 120 kV, attached to a CCD camera and a scanning tunneling microscope (Agilent 5500). Electrical conductivity measurements of DC-type were examined by a Pyrex glass conductivity cell operated till 500 °C. The resistance measurements were measured by a Keithley 610C solid-state electrometer. Around 500 mg of the sample was placed between two electrodes (1.0 cm diameter) and pressed by the upper electrode in each run to ensure good contact between the particles. A WEMA temperature controller was used to control the measured temperature. More particularly, the gravimetric method was used to determine the water uptake of different epoxy coatings. The fabricated samples were immersed in 0.1 M potassium chloride solution for certain intervals of time. Electrochemical impedance (EI) spectroscopy was conducted using a potentiostat of type Auto lab PGSTAT30, coupled to a computer equipped with the FRA software. A three-electrode arrangement was used, consisting of an Ag/AgCl reference electrode, a platinum counter electrode and our modified EPYR/GNP/MWCNT_2–30_-coated stainless steel electrodes (exposed surface area 3 cm^2^ and 100 μm thickness layer) as working electrodes, which were immersed in 0.1 M potassium chloride solution. EIS measurements were conducted potentiostatically at open circuit potential (*E*_cor_) with 10 mV rms with frequency range of 50 kHz to 0.1 Hz.

## 3. Results and discussion

Carbon-based materials are used by many researchers as desirable reinforcement agents that can be easily reinforced in different matrices, and their assistance in enhancing various properties of different polymer matrices has been studied as well. Such materials are widely evaluated due to their extensive as well as excellent electrical, thermal, mechanical as well as tribological properties. More particularly, carbon-based materials are favorable candidates for the reinforcement of a variety of polymer matrices. Considerable research papers have previously elucidated the positive impact of different forms of CNTs as well as GNPs separately on the pure epoxy resins except our previous paper, in which we optimized the mixing ratio of both CNTs and GNP.^23^ Therefore, as a continuation of our previous study, a new set of EPYR/GNP/MWCNT_2–30_ composite materials based on variable loadings of mixed GNP/MWCNT is fabricated by ultrasonic support. The fabricated products are characterized by common techniques. In addition, the whole enhanced performance of the neat epoxy resin is investigated by thermal analyses, electrochemical impedance and water sorption. A significant interest is given to the real enhancement in the coating behavior, which is observed for the neat epoxy resin due to different loadings of mixed GNP/MWCNT in the form of our targeted composite materials.

### 3.1. EPYR/GNP/MWCNT_2–30_ characterization and identification techniques

**Fig. 1:**
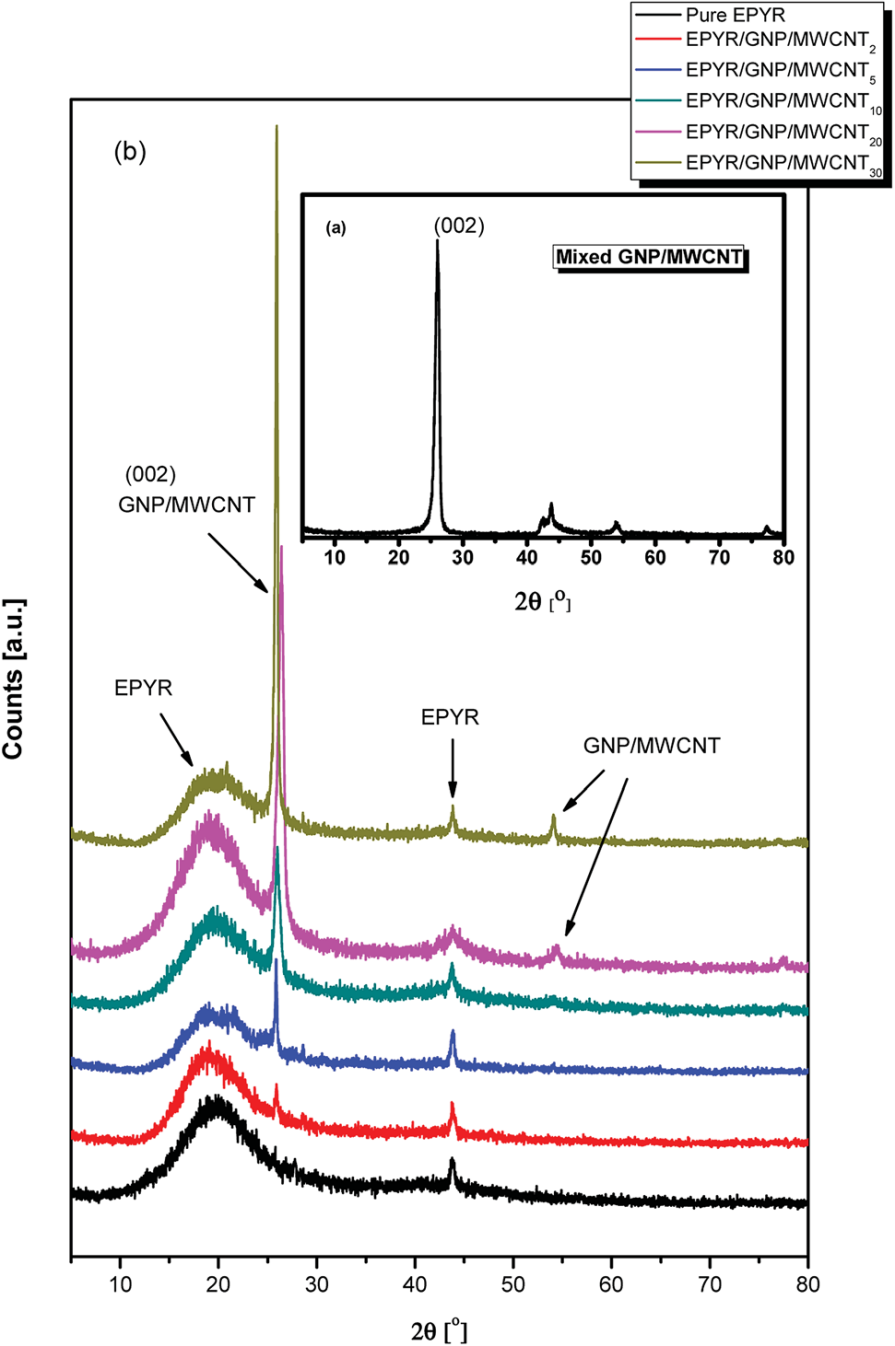
XRD patterns of (a) mixed GNP/MWCNT, (b) pure EPYR and its corresponding EPYR/GNP/MWCNT_2–30_ composite materials.

XRD analyses of neat epoxy, mixed GNP/MWCNT and their variable loaded EPYR/GNP/MWCNT_2–30_ composite materials are investigated in 2*θ* range from 5 to 80°, as illustrated in Fig. 1a and b. An excellent evidence for the formation of our composite materials is given by XRD diffraction patterns throughout the fabrication process. Carbon-based nano-fillers (GNP/MWCNT) show a typical XRD diffraction pattern for GNP and/or MWCNT, as shown in Fig. 1a. Fig. 1b shows the typical XRD patterns for neat epoxy and its corresponding EPYR/GNP/MWCNT_2–30_ composite materials; these patterns are in agreement with previously reported results for both neat epoxy and GNP/MWCNT.^23–32^Fig. 1a shows four important diffraction peaks at 2*θ* = 26.25°, 42.90°–44.8°, 53.98° and 77.32°, which match with those reported for any carbon-based nano-fillers.^26–29^ The first peak is the most important characteristic peak for carbon-based materials, which is assigned to the graphite plane (002). Fig. 1b shows characteristic peaks for amorphous EPYR, and they are assigned to its amorphous nature. The first broad as well as strong peak is observed in the range of 2*θ* = 10.5°–30.82°. However, other amorphous peaks have weak intensities, and they are located in the range of 2*θ* = 43.5°–45.3°.^23–25,30–32^ Furthermore, diffractograms in Fig. 1b also show the diffraction patterns for EPYR/GNP/MWCNT_2–30_ composite materials. From the first look, it is easy to confirm the composite formation, and the most important characteristic peaks for both neat epoxy and mixed GNP/MWCNT are easily detected in these patterns. The XRD diffractogram of EPYR/GNP/MWCNT_2_ (which contains 2 wt% of nano-filler loading) is similar to that of neat epoxy; this observation corresponds to the low nano-filler loading.^33^Fig. 1b shows broad peaks in the range of 2*θ* = 10.65°–30.85°, which are due to neat EPYR; the strong intensity peaks at around 2*θ* = 26.22° (002) are due to the graphite panel of carbon-based mixed nano-fillers because of the significant increase in the mixed filler content. A closer view of Fig. 1b displays similar peaks without any 2*θ* shift. Moreover, the peaks at around 2*θ* = 26.22° (002) for mixed GNP/MWCNT considerably increase with an increase in its loading ratios inside the composite materials. Meanwhile, the patterns for these composite materials still show the main characteristic peak for pure EPYR with additional loading in the mixed fillers. Previously reported data confirm that during the fabrication process, the structures of both nanofiller and/or epoxy matrix are intact without any cracking. No other crystalline peaks that may be assigned to other products or impurities are observed.

**Fig. 2:**
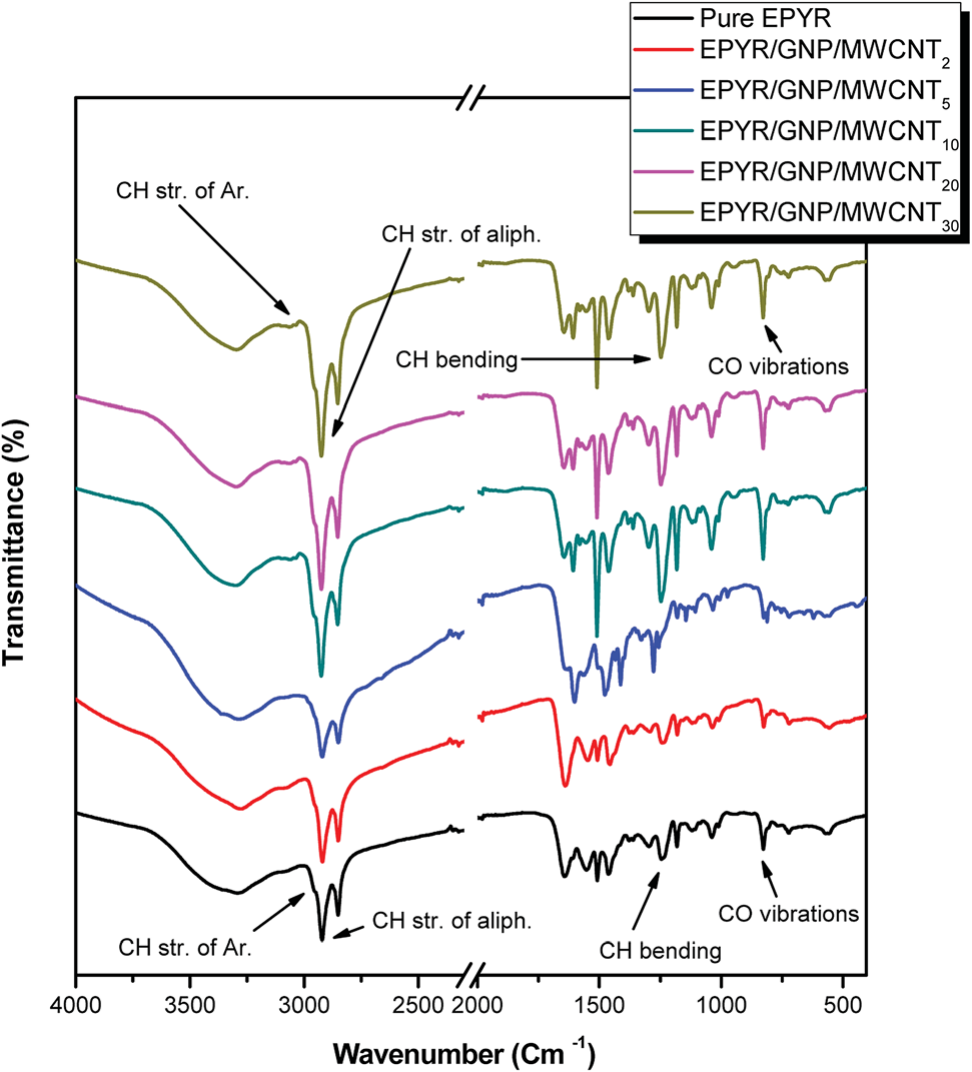
FT-IR spectra of pure EPYR and its corresponding EPYR/GNP/MWCNT_2–30_ composite materials.

FT-IR spectra for neat EPYR and its carbon-based EPYR/GNP/MWCNT_2–30_ composite materials represent another elucidation for the designed product fabrication, as displayed in Fig. 2. The predictable bonding interaction between EPYR and its reinforced GNP/MWCNT mixed nano-filler is explained. FT-IR results are examined by ATR smart part over the range of 400–4000 cm^−1^. FT-IR spectra show all important characteristic absorption peaks that are related to neat EPYR and mixed nanofillers, as reported in our previous study and literature.^23–25,34–39^ The peaks in the range of 3050–3015 cm^−1^ and at 3045 cm ^−1^ and 2965 cm^−1^ correspond to the valence CH vibrations of the epoxy ring, the stretching CH vibrations of the aromatic ring, and the stretching vibrations of the –CH_2_ functional group, respectively. Other peaks in the range of 930–815 cm^−1^ and at 1245 cm^−1^ are assigned to the valence CO vibrations of the epoxy ring and the bending CH vibrations of the epoxy ring, respectively. The neat EPYR characteristic peaks are also observed in the FT-IR spectra of EPYR/GNP/MWCNT_2–30_ composites. Moreover, the most relevant characteristic absorption peaks of carbon-based mixed nano-fillers are also observed in the composite material spectra (Fig. 2).^36–39^

**Fig. 3:**
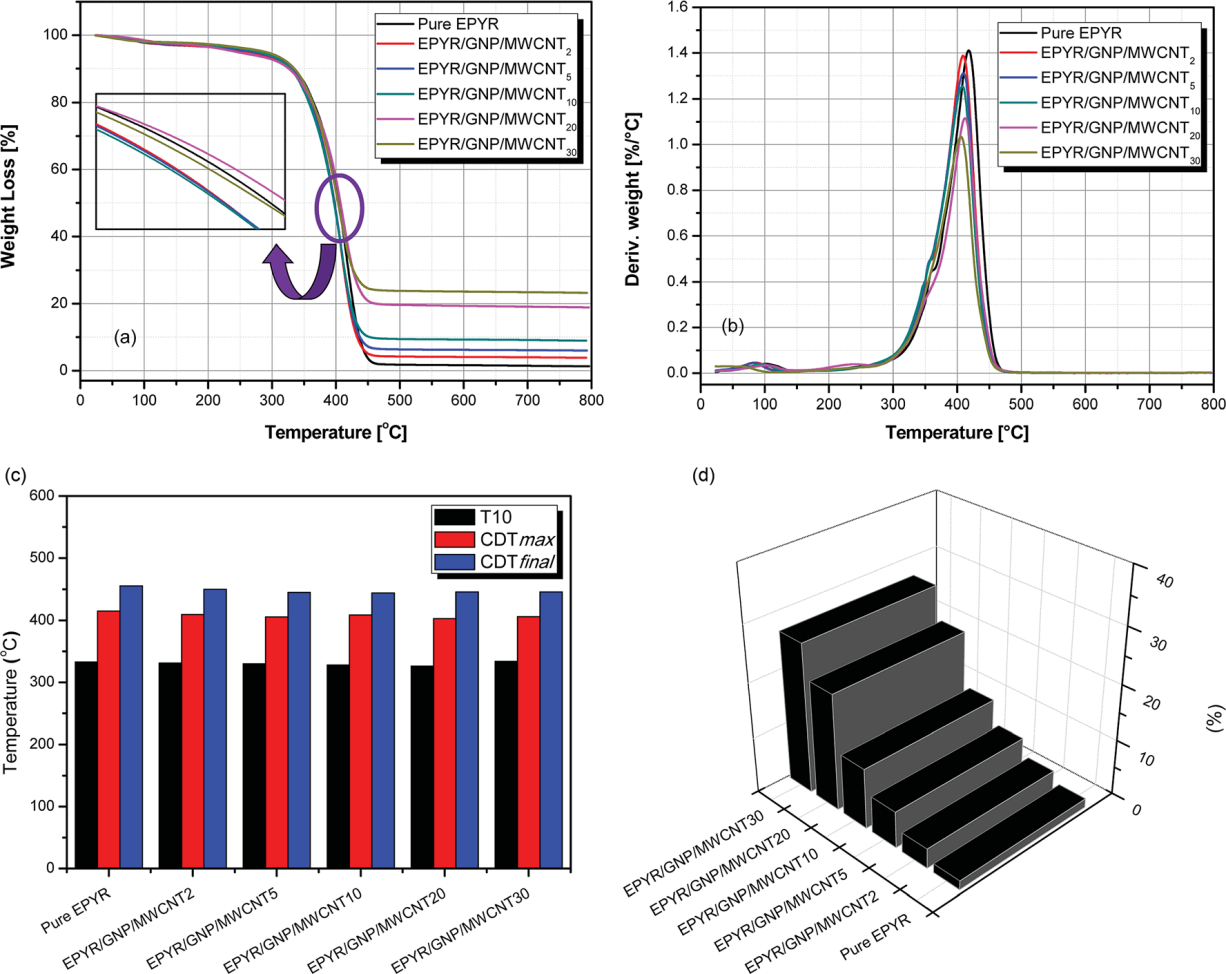
(a) TGA thermograms of pure EPYR and its corresponding EPYR/GNP/MWCNT_2–30_. (b) DTG thermograms of pure EPYR and its corresponding EPYR/GNP/MWCNT_2–30_. (c) *T*_10_, CDT_max_ and CDT_final_ temperatures for pure EPYR and its corresponding EPYR/GNP/MWCNT_2–30_. (d) Residue percent at 500 °C for pure EPYR and its corresponding EPYR/GNP/MWCNT_2–30_.

The thermal behaviour of pure EPYR and its related EPYR/GNP/MWCNT_2–30_ composite materials is studied by TGA & DTG analyses, as illustrated in Fig. 3a–d. It is clear from the TG curves that pure EPYR and EPYR/GNP/MWCNT_2–30_ nanocomposites are decomposed in one essential stage with similar behaviors for all materials. This step is detected in the range of 320–450 °C, and almost 80% weight loss is observed in this step, which results in the fast decomposition of all materials. Before attempting this step, a small weight loss is detected up to 110 °C, which mainly refers to the loss of entrapped solvents and moisture on the surface of these composite materials.^24,25^ In the range of 400–410 °C and during decomposition, pure EPYR and EPYR/GNP/MWCNT_20,30_ show higher thermal stabilities than other materials. EPYR/GNP/MWCNT_20_ shows remarkable increase in the thermal stability compared to previously mentioned materials. The order of increasing stability is EPYR/GNP/MWCNT_20_ > EYPR/EPYR/GNP/MWCNT_30_. The remaining materials, *i.e.*, EPYR/GNP/MWCNT_2–10_ show lower stabilities. The mixed nano-fillers (GNP/MWCNT) enhance the thermal decomposition of pure EPYR and act as catalysts, as illustrated in Fig. 3a. In addition, *T*_10_ amounts refer to the temperatures at which 10% weight losses are examined, as recorded in Table 2 and Fig. 3c. Pure EPYR and its related EPYR/GNP/MWCNT_2–30_ display similar thermal stabilities at this temperature (330 ± 4 °C), which means that the mixed nano-fillers have no direct effect on the thermal stability of neat EPYR at *T*_10_. CDT_max_ represents the maximum temperature at which decomposition is detected.^23,28^ CDT_max_ values are determined from DTG curves, as shown in Fig. 3b and Table 2. All products and neat EPYR have CDT_max_ values in the range of 425–402.7 °C. Neat EPYR shows the highest value compared to other products. CDT_max_ values also display gradual decrease with the increasing nano-filler content except the value for EPYR/GNP/MWCNT_30_. The final composite degradation temperature can be abbreviated as CDT_final_, and it indicates the final temperature at which decomposition is complete.^40,41^ CDT_final_ values are determined from the TGA curves, as recorded in Table 2 and Fig. 3c. The CDT_final_ values also range from 455.8 °C to 444 °C. A similar result is also observed: a significant decrease is observed for all samples by increasing the nano-filler content. It is clearly observed from Fig. 3c that the mixed GNP/MWCNT nano-fillers have no significant effect on *T*_10_, CDT_max_ and CDT_final_ temperatures for pure EPYR. *R*_500_ represents the remaining solid residues at 500 °C for neat EPYR and its related products, as illustrated in Fig. 3d; 1.6% residue is detected from EPYR, which refers to the final decomposition product of epoxy resin in the form of char and other materials. *R*_500_ values also show an expected percentage increase with the increase in mixed filler loading; subsequently, EPYR/GNP/MWCNT_30_ displays the highest residual value, and EPYR/GNP/MWCNT_2_ displays the lowest value. *R*_500_ values for the other products are almost in agreement with the loading contents of mixed nano-fillers with respect to the residual part for pure EPYR. An easy way to understand this behavior is given in the experimental section.

**Table 2:** Thermal behavior of pure EPYR and its corresponding EPYR/GNP/MWCNT_2–30_ composite materials

Abbreviation	*T*_10_ a (°C)	CDT_max_^b^ (°C)	CDT_final_^a^ (°C)	*R*_500_ (%)
Pure EPYR	333	415	455.8	1.6
EPYR/GNP/MWCNT_2_	331	409.3	450	3.49
EPYR/GNP/MWCNT_5_	330	405.6	445	6.55
EPYR/GNP/MWCNT_10_	328	408.5	444	10.84
EPYR/GNP/MWCNT_20_	326	402.7	446	20.89
EPYR/GNP/MWCNT_30_	334	405.9	446	26.9

a Values were determined by TGA thermograms at heating rate of 10 °C min^−1^

b Values were determined by DTG thermograms.

**Fig. 4:**
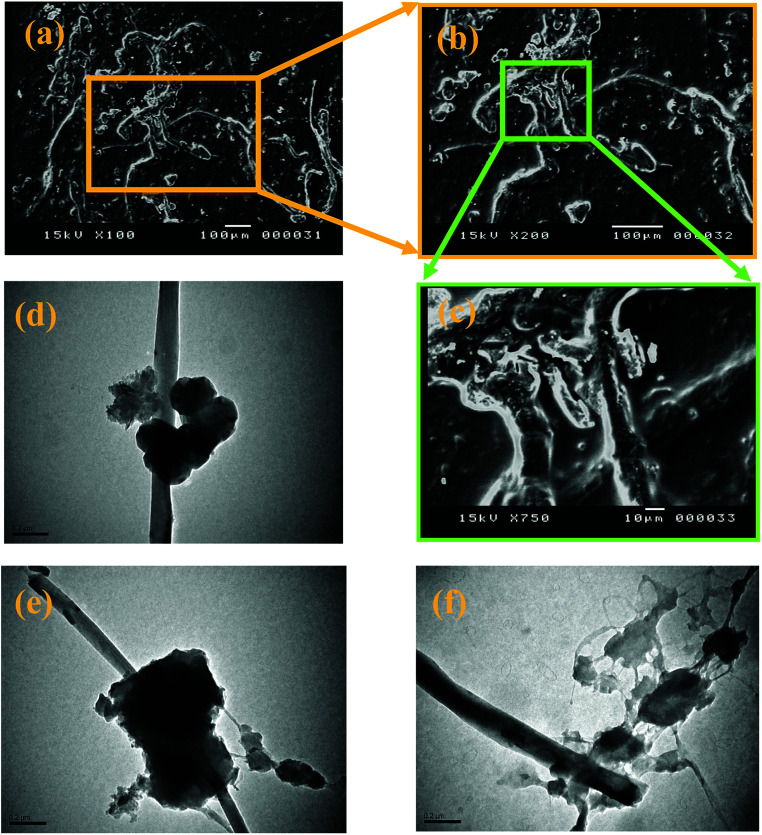
SEM images of EPYR/GNP/MWCNT_10_ (a–c) at magnifications of *x* = 100, 200 and 750, respectively; TEM images for EPYR/GNP/MWCNT_10_ (d–f).

The morphological studies of neat RPYR and its related EPYR/GNP/MWCNT_2–30_ products are examined by SEM and TEM instruments, as illustrated in Fig. 4. SEM measurements are essentially focused on the surface morphological changes that may be found upon reinforcing the EPYR matrix with carbon-based nano-fillers, as given in Fig. 4a–c. A dilute solution of each tested compound has been prepared and allowed to undergo an evaporation process on a smooth surface of aluminum foil followed by coating with gold palladium alloy. A Pentax Z-50P camera with Ilford film is used to obtain the required images at an accelerating voltage of 15 kV with nearly 4 mm work distance carbon film using the low dose technique.^42^Fig. 4a–c show the surface morphology of EPYR/GNP/MWCNT_10_ with an insertion of mixed GNP/MWCNT nano-fillers equal to 10% at magnifications of *x* = 100, 200 and 7500. It is clearly noticed from Fig. 4a–c that the mixed nano-fillers are distributed well, and they exhibit compatibility as well as strong adhesion as a result of nanocomposite formation between neat EPYR and reinforced nano-fillers. This observation indicates excellent miscibility between organic polymer matrix in the form of EPYR and its reinforced inorganic nano-fillers in the form of mixed GNP/MWCNT. Moreover, no particle agglomeration or cluster formation is observed in any image, which also confirms the uniform distribution of these products.

More particularly, another morphological characteristic is detected by TEM images. Fig. 4d–f represent the TEM micrographs of the same nanocomposite at magnification of 0.2 μm. With reference to the variable electron beam penetrability, Fig. 4d and e show that the imbedded mixed nano-fillers are clearly observed in the nano-platelet and nanotube structures, and the nanocomposite components are extremely compatible. In addition, no significant assemblage is observed.

**Fig. 5:**
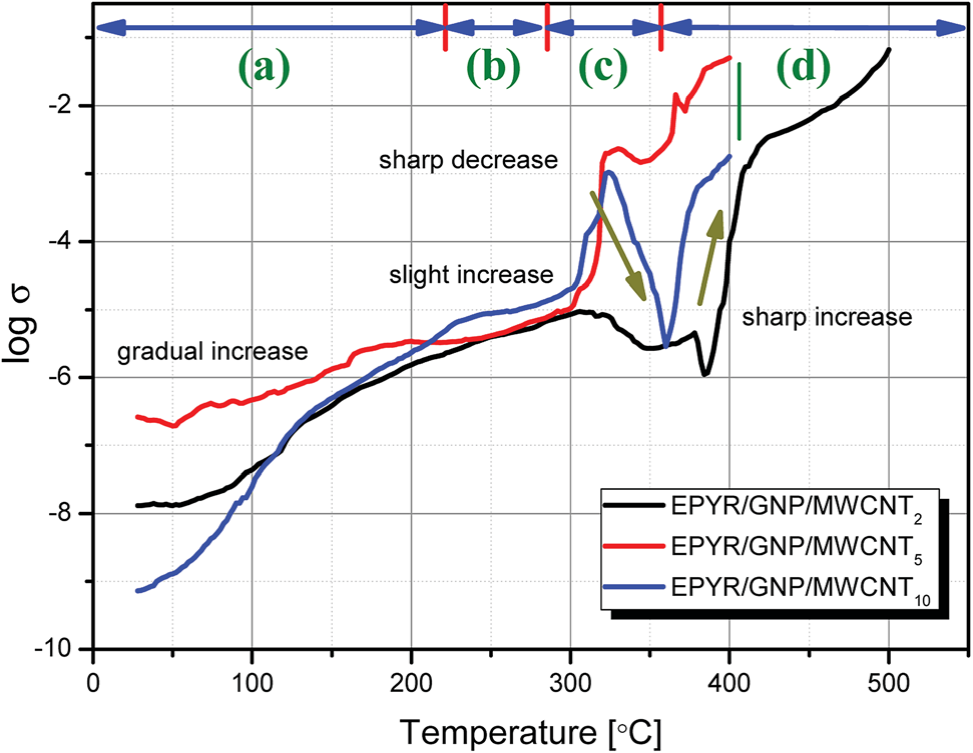
*In situ* electrical conductivity measurements for pure EPYR and its corresponding EPYR/GNP/MWCNT_2–10_ composite materials, at a heating rate of 10° min^−1^.

In addition, *in situ* electrical conductivity study demonstrates remarkable character detection of the change in phases during heating of pure as well as hybrid materials. The experiments are carried out for EPYR/GNP/MWCNT_2–30_ nanocomposites in the temperature range of 25–500 °C. The relation between log *σ* and temperature is illustrated in Fig. 5 for EPYR/GNP/MWCNT_2–10_. Measuring the conductivities for EPYR/GNP/MWCNT_20,30_ is quite difficult due to the extremely high conductivity values obtained in both cases, which are not within reasonable conductivity range of our instrument. This is due to the higher content of mixed GNP/CNT nano-filler loading in the epoxy matrix. As reported in the literature, conductivity values increase upon reinforcement of pure epoxy sample with nano-fillers due to the formation of the novel organic/inorganic nanocomposite formation.^43–45^ Hence, the conductivity values for EPYR/GNP/MWCNT_2–10_ at room temperatures (28 °C) are 1.3 × 10^−8^, 2.6 × 10^−7^ and 7.3 × 10^−10^ Ω ^−1^ cm^−1^. Upon increasing the temperature, the conductivity values considerably increase. At 200 °C, *σ* values are 1.5 × 10^−6^, 3.4 × 10^−6^ and 2.3 × 10^−6^ Ω ^−1^ cm^−1^ for these compositions. Finally, more enhanced conductivity is observed at 400 °C, where the values reach the conducting region (1 × 10^−4^, 4.9 × 10^−2^ and 1.8 × 10^−3^ Ω ^−1^ cm^−1^). Therefore, the conductivity values display that EPYR/GNP/MWCNT_5_ has the best conductivity among the three measured samples over the measured temperature range. Moreover, the results in Fig. 5 are divided into four categories (a–d). A gradual increase in the conductivity along with the increasing temperature is mentioned in the 1^st^ region from room temperature to 220 °C; EPYR/GNP/MWCNT_5_ also shows the best conductivity value at this period. A slight increase is observed in the 2^nd^ region in the range of 220–285 °C. The content of nano-filler loading highly affects the conductivity values, and the order of increasing *σ* values is EPYR/GNP/MWCNT_10_ > EPYR/GNP/MWCNT_5_ > EPYR/GNP/MWCNT_2_. The 3^rd^ region is located in the range of 285–355 °C. In this region, again, a strong sharp increase followed by intense decrease is seen, which may be due to the starting of decomposition of these materials and hence, there is no more epoxy matrix. A sharp increase is still observed in the fourth region from 355–500 °C. Moreover, the results of 3^rd^ and 4^th^ regions confirm that the order of increasing conductivity does not depend on the loading of these carbon-based nano-fillers. EPYR/GNP/MWCNT_5_ again shows the best conductivity values in these regions. This suggests that EPYR/GNP/MWCNT_5_ represents the critical or optimal composition that causes synergic conductivity enhancement over this temperature range.

### 3.2. Promoted epoxy coating performance

The coating performance of laminating resins of pure EPYR is a very important character, which is applied over a wide range of applications from thin film epoxy coatings to fabricated fiber reinforced composites. Epoxy resin-controlled designed fabrications produce epoxy compounds with superior characters with respect to an industry leading materials. Nano-filler addition is reported as an important modification of epoxy resins. Therefore, the enhanced epoxy coating performance has been utilized in the sorption of water (SW) experiment and electrochemical impedance (EI) spectroscopic technique, which is considered as a typical technique for detecting the coating efficiency of our fabricated materials.

**Fig. 6:**
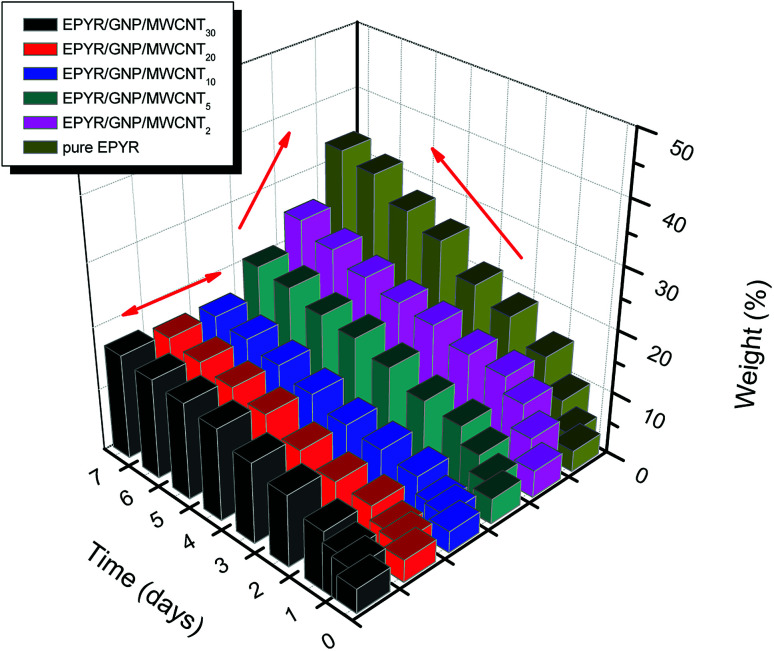
Sorption of water for pure EPYR and its corresponding EPYR/GNP/MWCNT_2–30_ composite materials (immersion time in days).

Researchers always perform such SW experiments to obtain preliminary indication about the actual efficiency of any material. The experiment mainly depends on the ability of each sample to gain a specific amount of water when it is immersed in a solution of 0.1 M potassium chloride for certain intervals of time at room temperature using the recognized gravimetric technique. The results of the SW experiment for neat EPYR and its corresponding EPYR/GNP/MWCNT_2–30_ after immersion time up to 7 days are given in Fig. 6. The given masses are calculated according to the following equation:

1 WS = (*M*_*t*_ − *M*_o_)/*M*_o_ × 100.

Here, *M*_o_ represents the mass before immersion and *M*_*t*_ represents the mass after immersion for each time. For the first few days, the measurements are very fast and then, once a day is sufficient.

The neat epoxy sample shows continuous increase in the calculated mass during the experiment as it may have very large values of SW. A sharp line can be obtained from the graph after 2 days due to the larger amount of water gained; then, a straight line can be formed, indicating the gradual increase up to the end. A continuous swelling property of EPYR is clearly observed as a result of higher rate of water immersion.^46,47^ A similar result has been obtained for EPYR/GNP/MWCNT_2,5_ with respect to the lower amount of water gained in each case. EPYR/GNP/MWCNT_2_ shows the poorest water absorption capability due to its lower nano-filler loading. EPYR/GNP/MWCNT_10–30_ nanocomposites show different behaviors, and slight increase can be observed with constant mass, which is due to the limited amount of absorbed water in these compositions because of the higher loading of mixed GNP/MWCNT nano-fillers; this efficiently suppresses the SW process. The mixed fillers strongly hinder water uptake because all available unoccupied holes inside the EPYR are closed. SW noticeably decreases with the increasing nano-filler loading up to EPYR/GNP/MWCNT_10_, which shows the best water prevention composition; then, the value remains almost constant.

**Fig. 7:**
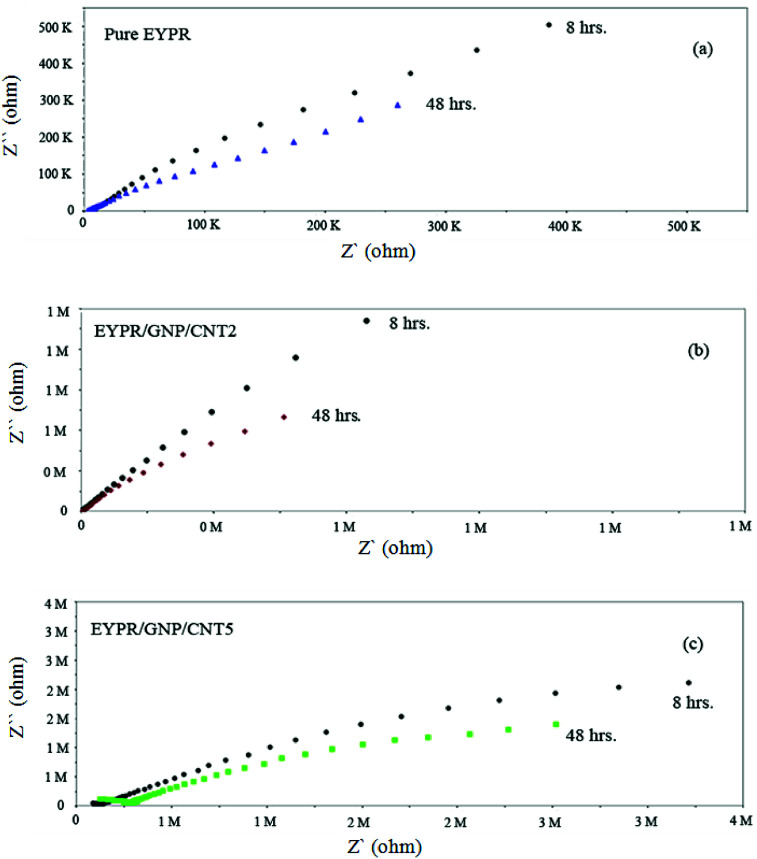
Electrochemical impedance spectra of pure EPYR (a) and EPYR/GNP/MWCNT_2,5_ (b and c) after immersion time of 8 and 48 hours.

**Fig. 8:**
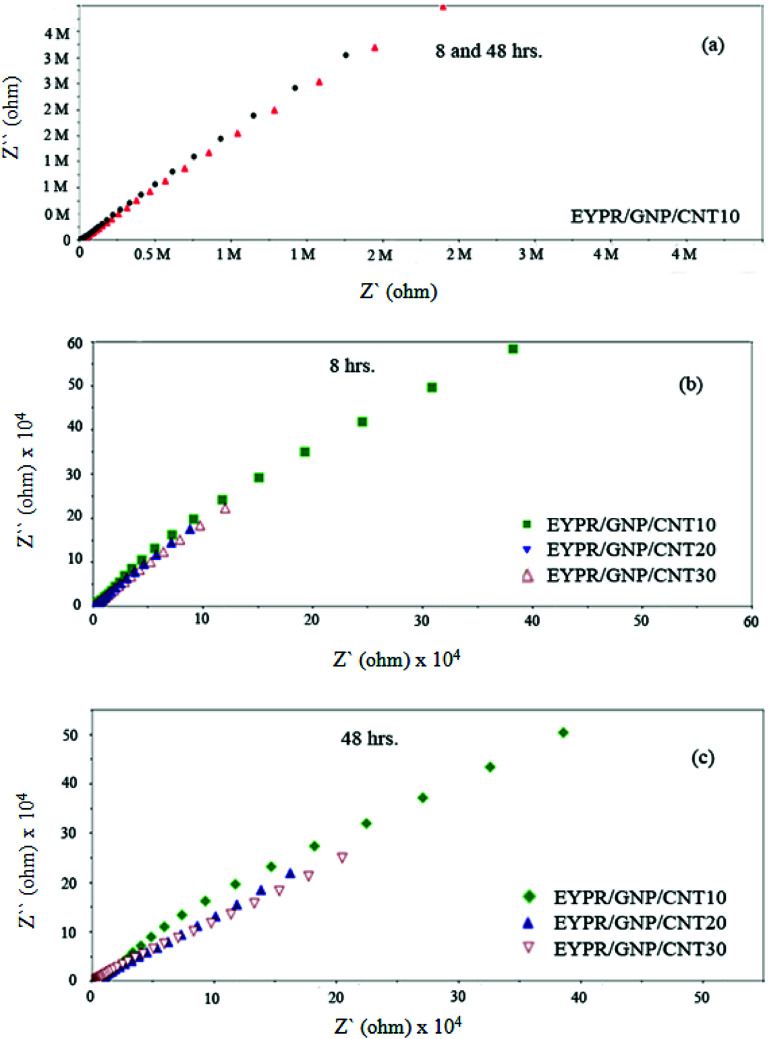
Electrochemical impedance spectra of EPYR/GNP/MWCNT_10_ after immersion time of 8 and 48 h (a), EPYR/GNP/MWCNT_10–30_ after immersion time of 8 h (b) and EPYR/GNP/MWCNT_10–30_ after 48 h (c).

**Fig. 9:**
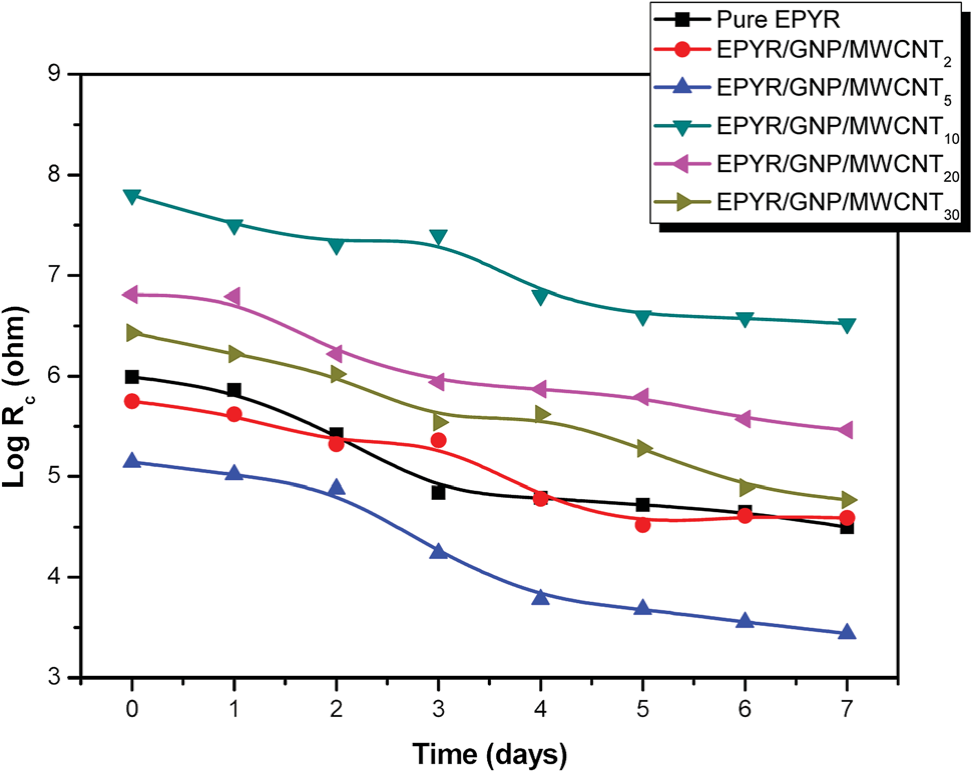
Change of *R*_c_ in accordance with the immersion time for pure EPYR and its corresponding EPYR/GNP/MWCNT_2–30_ composite materials.

Furthermore, an enhanced coating performance for EPYR through nanocomposite formation in the form of EPYR/GNP/MWCNT_2–30_ has been further confirmed by EI spectroscopy. Electrode fabrications over stainless steel substrates are described in the experimental section. A solution of 0.1 M potassium chloride is used during the measurements for 168 hours. A Nyquist plot shows the final impedance curves representing the obtained results as persistent measurements of each fabricated electrode at calculated fixed times.^48,49^ The Nyquist plots obtained from EI measurements for EPYR and its related EPYR/GNP/MWCNT_2–30_-coated steel samples after 8 and 48 hours immersion times are shown in Fig. 7 and 8, respectively. All fabricated materials show higher coating resistance than pure EPYR although EPYR/GNP/MWCNT_2,5_ exhibit similar behaviors due to the lower content of mixed nano-filler loading. Fig. 7a–c display the impedance coating spectra of EPYR, EPYR/GNP/MWCNT_2_ and EPYR/GNP/MWCNT_5_. This behavior is controlled by the coating capacitance at high frequencies and coating resistance at low frequencies. Fig. 7a–c show significant decrease in the coating resistance after 48 h immersion, which refers to the water immersion inside EPYR. Such an observation is also detected from SW experiment as previously discussed. Such motion of ionic species inside pure EPYR arises basically due to large water content, which enhances the conductivity of the coated layer.^49,50^Fig. 8a shows that typical enhanced epoxy coating behaviour is represented by the impedance coating spectra of EPYR/GNP/MWCNT_10_. Extremely stable coating resistance values are achieved after 8 and 48 hours immersion times compared to the values of EPYR and its related EPYR/GNP/MWCNT_2,5_. Such monitoring mainly refers to lower SW in this composition. Mixed GNP/MWCNT efficiently suppresses the SW process and therefore increases the coating resistance and the coating capacitance, as described in SW section. Comparable results have been observed for EPYR/GNP/MWCNT_20,30_, which also refer to the presence of sufficient amount of nano-fillers for preventing the SW process; this is also in accordance with the results of the gravimetric experiments. Fig. 8b and c show comparative Nyquist plots of EPYR/GNP/MWCNT_10–30_ after 8 and 48 hours, respectively. It is clearly seen that there is no significant change in the coating resistance between these three nanocomposites after 8 hours, whereas an inconsiderable decrease is observed after 48 hours. Moreover, the coating resistance variations (CRv) for EPYR and its EPYR/GNP/MWCNT_2–30_ as a function of increase in the immersion time are illustrated in Fig. 9. The results are in accordance with those discussed for the gravimetric method and EI studies. The CRv values of all compositions show slight decrease with the increasing immersion time. However, a slight decrease and/or remaining constant after 168 hours of immersion is observed for EPYR/GNP/MWCNT_10_. It is clearly observed that the CRv values of EPYR/GNP/MWCNT_10_ are the highest values among the values for the measured compositions, closely followed by those of EPYR/GNP/MWCNT_20_ and EPYR/GNP/MWCNT_30_. Both EPYR/GNP/MWCNT_2,5_ display lower CRv values than that obtained for pure EPYR. The main reason for CRv values' decrease is the considerable increase in the coating conductivity due to immersion of water ions inside the coating materials.^49^ In conclusion, the mixed GNP/MWCNT nano-fillers cause remarkable improvement in the EPYR coating, which may be due to the ionic charge transfer resistance and elevated barrier behaviour. These mixed nano-fillers effectively enhance the coating behaviour of EPYR than other reported nanofillers.^23–25^

## 4. Conclusions

Carbon-based nanofillers in the form of GNP/MWCNT composites were utilized as reinforcement agents during the fabrication of new category of epoxy resin composite materials by ultrasonic support. Variable loadings of these nano-fillers were introduced while preparing the epoxy resin. The final products were characterized by common characterization tools including XRD diffraction, FT-IR, SEM and TEM tests. Moreover, the impact of these nano-fillers on the total efficiency of pure EPYR was examined by thermal analyses, electrical conductivity, sorption of water and electrochemical impedance measurements. All products and neat EPYR exhibited CDT_max_ values in the range of 425–402.7 °C. Neat EPYR showed the highest value compared to the other products. CDT_max_ values also displayed gradual decrease with the increasing nano-filler content except for the value of EPYR/GNP/MWCNT_30_. CDT_final_ values were also in the range from 455.8 °C to 444 °C. The thermal results clearly indicated that the mixed GNP/MWCNT nano-fillers have no significant effects on *T*_10_, CDT_max_ and CDT_final_ temperatures for pure EPYR; on the contrary, significant effects on the *in situ* electrical conductivity measurements were observed. The conductivity values reached the maximum and could not be measured for EPYR/GNP/MWCNT_20_,_30_ due to their higher contents of mixed nano-fillers. Nyquist plots for pure EPYR, EPYR/GNP/MWCNT_2_ and EPYR/GNP/MWCNT_5_ showed similar behavior; on comparison, we found that EPYR/GNP/MWCNT_20,30_ exhibited quite good coating results. CRv values of EPYR/GNP/MWCNT_10_ were the highest among the values of the measured compositions, closely followed by those of EPYR/GNP/MWCNT_20,30_. Both EPYR/GNP/MWCNT_2,5_ displayed lower CRv values than that obtained for pure EYPR.

## Conflicts of interest

There are no conflicts to declare.

## Supplementary Material
